# Open surgical management of ureteric strictures: A retrospective study

**DOI:** 10.6026/973206300210405

**Published:** 2025-03-31

**Authors:** Sanjay P. Dhangar, Dhaval Rasal, Shashank Patil, Amirtha Balakumar, Swapnil Vaidya

**Affiliations:** 1 Department of Urology Bharati Hospital & Research Centre, BVU, Pune, Maharashtra, India; 2 Department of General Surgery & Urology, Melmaruvathur Adiparasakthi Institute of Medical Sciences (MAPIMS), Melmaruvathur, Tamil Nadu, India; 3 Department of Urology, VishwaRaj Hospital, Pune, Maharashtra, India

**Keywords:** Ureteric stricture, ureteric reconstruction, buccal mucosal ureteroplasty, peritoneal flap reconstruction, ileal ureter

## Abstract

Ureteral strictures may arise from long standing nephro/urolithiasis, radiation, use of lasers for treatment of stones, trauma, ischemia, and iatrogenic injury. Therefore, it is of interest to assess the long-term results of ureteral reconstruction using different techniques. Hence, all benign ureteral strictures that more than 2 cm at the level of the pelvi-ureteric junction, upper or mid-ureter, or loss of long length of ureter not suitable for Boar's flap or ureteric reconstruction at a tertiary hospital were included in this study. The mean length of the stricture was 4.5 cms with longest stricture length of 10.4 cms. The success rate of the ureteric reconstruction surgery in our study was 96.7% with recurrence in 3.2% cases after 13 months. Thus, the largest study on open ureteric reconstruction using variety of techniques is reported.

## Background:

Ureteral strictures may arise from long standing nephro/urolithiasis, radiation, use of lasers for treatment of stones, trauma, ischemia, and iatrogenic injury [1]. The treatment options for ureteral strictures are wide depending upon the location of the stricture. Ureteric reconstruction is associated with good long-term results with minimal morbidity [2, 3]. Minimally invasive surgical treatment for ureteral strictures includes balloon dilatation, catheter dilatation and endoureterotomy with laser or bug bee electrode. However, long-term efficacy of these procedures is not known because of the retrospective nature of the studies and short-term follow-up [4, 5, 6, 7-8]. Ureteric re-implantation and Boar's flap techniques can be used for lower ureteric strictures [9] while strictures of mid and upper ureter are difficult to treat with these techniques. Small strictures can be treated by uretero-ureterostomy or pyeloplasty, wherever possible. Infact, complex/long ureteric strictures which are not amenable to the above-mentioned techniques pose a clinical challenge to the urologist. These strictures can be treated by appendiceal interposition, ileal ureter replacement, buccal mucosal graft, peritoneal flaps or renal auto transplantation [10]. The European Association of Urology (EAU) guidelines for urological trauma have also specified oral graft for the treatment of long segment ureteric stricture [11]. A meta-analysis done by You *et al.* showed superior results of oral mucosa when compared with the intestinal interposition [12]. Oral mucosa is resistant to infection, compatible with a wet environment and is hairless. Moreover, it has a thin lamina propria and thick epithelial layer that facilitates the imbibition and inosculation [13]. Therefore, it is of interest to assess the long-term results of ureteral reconstruction using different techniques.

## Materials and Methods:

## Study design and patient selection:

This study is a single-center retrospective study. All patients from August 2016 to July 2023 were included in the study. All patients underwent open ureteric reconstruction. The study was approved by the institutional ethical committee of the SMBT IMS and RC, Nashik and Maharashtra. The indication for ureteric reconstruction was a benign ureteral stricture more than 2 cms at the level of the pelvi-ureteric junction, upper or mid-ureter, or loss of long length of ureter not suitable for Boar's flap or ureteric reconstruction.

## Inclusion criteria:

[1] Age ≥ 18 years

[2] Stricture ≥ 2 cms

[3] Written and informed consent for open surgery

## Exclusion criteria:

[1] Active UTI (urinary tract infection)

[2] Smaller/Malignant ureteric stricture

[3] Uncorrectable coagulopathy

[4] Patient or relatives not willing for open surgery

## Preoperative preparation:

All patients were admitted. Double J stenting or nephrostomy was done to drain the system and decrease the hydro/pyonephrosis. A preoperative retrograde/antegrade pyelography was performed in all the cases to confirm the length and location of the stricture. Renal function was confirmed by radionuclide imaging in the patients where there was doubt of poor functioning after CT urography. Urine culture was done in all patients preoperatively. Patients with significant bacteriuria (≥100,000 CFU/mL) were treated peri-operatively with the specific antibiotics.

## Surgical technique:

All patients were catheterised and the catheter was kept in the drapings in sterile field. We used extra-peritoneal approach. Flank incision was taken in cases of pelvi-ureteric junction and upper/mid ureteric strictures in lateral flank position. A midline laparotomy incision was taken in cases planned for appendiceal or ileal ureteric replacement. After exposure ureter was identified and mobilised adequately. Ureter was incised ventrally with extension of the incision to one centimetre above and below the level of the stricture and confirmed by seeing the healthy pink mucosa/tissue. Lateral peritoneal wall flap was mobilised without opening the peritoneal cavity. When appendix or ileum was used, proper mesenteric flaps were mobilised. Appendiceal base was ligated and inverted with figure of eight suture. Ileo-ileal anastomosis was done with vicryl 3-0 suture after separating the ileal segment for reconstruction. Buccal mucosa was harvested from the either left or right side of the buccal cavity. The graft was approximately 1.5-2 cm wide and 4-5 cm in length depending upon the length of the stricture. The defect was closed with chromic catgut 3-0 suture. After the flap/graft harvestating, a 6/26 Fr double-J stent was inserted over the guidewire. The anastomosis of the flap/graft and ureter edges was performed with 4-0 PDS/vicryl absorbable continuous sutures. Omentum was wrapped where indicated, similarly the graft was covered by retroperitoneal or perirenal fat. A retroperitoneal tube drain was placed below the anastomosis and wound was closed in two layers. The drain was removed when the output was <50 ml per day. A micturating cystogram or an intravenous pyelography was done before catheter removal on day 21 post-operatively. The double-J stent was removed after 6 weeks. Patients were followed in the out-patient department at 5 days, two weeks, one month and then three monthly after discharge. Contrast study was repeated at 3 months and one year follows up to look for the recurrence of the stricture and later by the recurrence/absence of the symptoms.

## Statistical analysis:

We used SPSS software version 20 for data analysis. We used descriptive statistical analysis after collecting the patient demographic data, perioperative data, and follow up records.

## Results:

Patient characteristics are displayed in (Table 1 and Table 2). Of the total 31 patients who underwent ureteral reconstruction, six patients had history of failed previous surgery. One patient had uretero-ureterostomy (3.2%) and another underwent peritoneal flap reconstruction (3.2%). Rest had failed pyeloplasty (12.9%). The cause of stricture primarily was either long standing obstructed ureteric calculus or in similar cases use of high energy laser power for fragmentation of the stone (64.5%). Diabetes mellitus and hypertension were the most common comorbidities (32.2%). Most common location of the stricture was the upper ureter (64.5%), followed by the mid ureter and pelvi-ureteric junction. The mean length of the stricture was 4.5 cms with longest stricture length of 10.4 cms. All patients underwent pre-operative stenting or nephrostomy drainage except 5 (16.1%) patients as either they were not willing or they were taken for surgery immediately after the diagnosis of the disease. BMG ureteroplasty (48.3%) was the most common type of the ureteric reconstruction procedure (Table 3) followed by the peritoneal flap reconstruction (29%). The mean time of surgery was 166 minutes with maximum surgery time of 230 minutes. The average blood loss was 100.6 ml. One patient (3.2%) needed blood transfusion where we did ileal ureteric replacement with ileo-calycostomy at the lower pole of the kidney. This patient was alcoholic and had pelvi-ureteric junction transaction that went un-noticed till patient came to us with ascites and formation of renal pelvis-peritoneal fistula. The fistula was repaired during the surgery. There were few intra-operative complications (19.3%) that were managed intra-operatively. No intervention was needed in the post-operative period. There were minor post-operative complications (Clavien-Dindo I) in eleven patients (35.4%). One patient (3.2%) was readmitted within one month of the surgery due to sepsis (Clavien-Dindo II) and was managed conservatively successfully with intra-venous antibiotics and supportive treatment. The mean duration of hospital stay was 5.5 days with standard deviation of 1.98 days. The success rate of the ureteric reconstruction surgery in our study was 96.7%. One patient (3.2%) had recurrence of the symptoms after 13 months (peritoneal flap was done) and was diagnosed with stricture recurrence. He was stented later.

## Discussion:

Ureteric stricture disease poses a challenging entity to the urologist because of the wide spectrum of the modalities required for the treatment. Traditionally, open ureteric reconstruction has been considered the gold standard method due to the adequate space and exposure available in the retro-peritoneum without dealing with the bowels. Open ureteric reconstruction using buccal mucosa was first described in 1999 by Naude [14]. Since then, many case reports and case series have been reported. For long strictures of ureter, European Association of Urology guideline place oral mucosal graft as an option for intestinal replacement [11]. Our good success with the buccal mucosa is comparable with results of Engelmann *et al.* [15]. They had 92.9% success rate. Tabularised peritoneal flap offers a good and viable option for long strictures of ureter. Brandao *et al.* in their study on porcine model demonstrated that tabularised peritoneal flap is a feasible and reproducible option [16]. Chaudhari *et al.* in their case report also showed good ureteric reconstruction results with peritoneal flap [17]. They showed that the peritoneal flap developed and sustained good blood supply and the peritoneal mesenchymal cells differentiate into urothelial cells and myofibroblasts. Their only concern was the long term functional obstruction and its effect on renal function. We had stricture recurrence in one patient at 13 months with a success of 88.8% after using peritoneal flap.

Appendiceal flap is a very good option for the complex and long strictures of the proximal and mid right ureter. Melnikoff [18] was the first person who attempted appendiceal replacement of the ureter. Yarlagadda *et al.* successfully did first robotic assisted appendiceal interposition [19]. Since then, many case series and studies were done. Burns et al reported 83.3% success report with appendiceal interposition [20]. Komyakov *et al.* achieved success of 96.2% in their study [21]. We had success in all our four cases. The benefits of appendix include absorption of minimal urine, less chances of electrolyte disturbances, the peristaltic movements allow the urine to pass smoothly and the blood supply is better than the buccal mucosa due to the preservation of mesoappendix. Ileal interposition/replacement though, has many risks but provide a very good option in long, complex, and recurrent ureteric strictures. The success rate for ileal ureter replacement as reported by You *et al.* [12] in their meta-analysis is around 85.8 %. We did only three cases of ileal interposition and replacement with 100% success rate. The mean length of stricture was 4.5 cm. You *et al.* [12] in their meta-analysis of oral mucosal graft versus ileal ureteric replacement had mean ureteric stricture length of 6.77cm. They also reported the mean length of hospital stay of 7.97 days. We had mean hospital stay 5.5 days. Porpiglia *et al.* in their study concluded that to preserve the homolateral kidney function, sub-total/partial replacement of ureter with ileum is effective as well as a safe procedure [22]. Peeker *et al.* in his study summarized that the newer techniques of ileal ureteric substitution and replacement are promising with good results [23]. Roth *et al.* in their assessed the long term results of ileal ureteric substitution and concluded that ileal ureteric substitution is a versatile procedure with good long term results in well selected patients [24]. The limitations of this study are the retrospective nature, only center involved and a smaller number of patients. We need randomized controlled and multicentre studies to reach a better and final recommendation.

## Conclusion:

The largest study on open ureteric reconstruction using variety of techniques is reported. All these techniques are feasible with a good success rate. However, proper guidelines and randomised trials are required. Further, urologists should be encouraged to do these procedures using minimal invasive techniques.

## Ethics statement:

This study was approved by the Ethics Committee of SMBT Institute of Medical Sciences & Research Center, Dhamangaon, Nashik, Maharashtra, India. The patients provided written consent.

## Abbreviations:

EAU = European Association of Urology

UTI = Urinary tract infection

## Financial support:

None

## Figures and Tables

**Table 1:** Patient and disease variables

**Characteristic**	**Outcome**
Age, mean (Range)	48.5 (31-74)
Gender, n (%)	
Male	19 (61.2)
Female	12 (38.7)
BMI, mean (Range) Co-morbidity	22.4 (20-36)
DM	6 (19.3)
HTN	4 (12.9)
Post PTCA	2 (6.4)
Chronic alcoholism	2 (6.4)
Chronic renal failure Stricture location, n (%)	1 (3.2)
Upper ureter	20 (64.5)
Mid ureter	7 (22.5)
Pelvi-ureteric junction (PUJ) Laterality, n (%)	4 (12.9)
Right	13 (41.94)
Left	18 (58.06)
Length of Stricture (cm), mean (Range) Aetiology, n (%)	4.5 (2.0-10.2)
Ureteral calculi (long standing obstruction)	14 (45.1)
History of endoscopic laser lithotripsy, n (%)	6 (19.3)
Secondary pelvi-ureteric junction obstruction (PUJO)	4 (12.9)
GUTB	2 (6.4)
Congenital	1 (3.2)
Previous ureteral reconstruction, n (%)	
Ureteroureterostomy	1 (3.2)
Peritoneal flap reconstruction	1 (3.2)
Trauma	1 (3.2)
Iatrogenic during open surgery	1 (3.2)
Preoperative urinary drainage	
Double-J stent	21 (67.7)
Nephrostomy	5 (16.1)
None	5 (16.1)

**Table 2:** Intra-operative and post-operative variables

	**Mean**	**Range**
Duration of surgery (minutes)	166	130-230
Blood loss (ml)	100.6	70-350
	**Number**	**Percentage**
Transfusion (No of patients)	1	3.2
Intra-op complications		
Pleural injury	2	6.4
Peritoneal breach	2	6.4
Bowel injury	1	3.2
Cardiac arrhythmias/arrest	1	3.2
Post-op complications (short & long term)		
Haematuria		
Wound infection	5	16.1
Paralytic ileus	4	12.9
Urinary extravasation	1	3.2
Stricture recurrence	1	3.2
Sepsis	1	3.2
	1	1
Duration of hospitalisation (Days)	Mean	SD
	5.5	1.98

**Table 3:** Ureteric reconstruction techniques

**Type of Ureteric Reconstruction Technique**	**Number (%)**
BMG ureteroplasty	15 (48.3)
Peritoneal flap reconstruction	9 (29)
Appendiceal flap interposition	4 (12.9)
Ileal interposition	2 (6.4)
Ileal replacement	1 (3.2)
Total	31
